# Effectiveness and safety of Chinese herbal acupoint application in adult patients with fever and mild-to-moderate COVID-19: a multicenter, randomized, double-blind, placebo-controlled trial

**DOI:** 10.3389/fneur.2025.1577976

**Published:** 2025-06-19

**Authors:** Yicheng Liu, Jiaheng Shi, Xinting Liu, Zongchen Jiang, Benliang Zou, Rui Zhang, Qiuyan Li, Peili Wang, Chenhao Zhang, Jia Wang, Zhixi Zhang, Jiao Huang, Baojin Hua, Luqi Huang, Wensheng Qi

**Affiliations:** ^1^Guang'anmen Hospital, China Academy of Chinese Medical Sciences, Beijing, China; ^2^National Clinical Cardiovascular Disease Research Center of Traditional Chinese Medicine, Xiyuan Hospital, China Academy of Chinese Medical Sciences, Beijing, China; ^3^Wangjing Hospital, China Academy of Chinese Medical Sciences, Beijing, China; ^4^Eye Hospital, China Academy of Chinese Medical Sciences, Beijing, China; ^5^Institute of Chinese Materia Medica, China Academy of Chinese Medical Sciences, Beijing, China

**Keywords:** traditional Chinese herbal, COVID-19, acupoint application, antipyretic effect, relief symptom

## Abstract

**Background:**

Chinese herbal acupoint application (HAA) is recommended by certain guidelines for treating mild-to-moderate COVID-19; however, evidence supporting its effectiveness remains limited. This study aimed to evaluate the effectiveness and safety of HAA in adult patients with fever and mild-to-moderate COVID-19.

**Methods:**

This multicenter, randomized, double-blind, placebo-controlled trial was conducted at six hospitals in China. Overall, 364 participants were randomly assigned in a 1:1 ratio to receive either the herbal or placebo acupoint application. All participants received applications at the Dazhui (GV14) and Feishu (BL13) acupoints three times daily for 2 h per application over 5 days and *Fuzheng Jiebiao Decoction* orally three times daily, three bags per dose. The primary outcome was complete fever relief time. Secondary outcomes included the onset time of fever reduction, changes in symptom scores, routine blood tests, and acetaminophen usage rates and dosages.

**Results:**

Regarding the primary outcome, HAA significantly reduced complete fever relief time compared to placebo (31.75 vs. 52.00 h; *p* < 0.0001). Regarding secondary outcomes, the herbal group also demonstrated a shorter onset time of fever reduction than the placebo group (24.35 vs. 34.42 h; *p* < 0.0001). HAA significantly reduced total symptom scores, particularly fever, headache, and cough symptoms. Moreover, 52 patients (29.05%) in the herbal group used acetaminophen, with a median dosage of 0.3 g (0.3, 0.6), which was significantly lower than that in the placebo group, with 94 patients using 0.6 g (0.3, 0.9; *p* < 0.05). No significant differences were observed in routine blood test results between the groups (*p* > 0.05), and no serious adverse events (SAEs) were reported in either group.

**Conclusion:**

Chinese herbal acupoint application effectively and safely shortened the complete fever relief time and onset time of fever reduction; alleviated clinical symptoms, particularly fever, headache, and cough; and reduced the need for antipyretic analgesics in adult patients with fever and mild-to-moderate COVID-19.

**Clinical trial registration:**

https://www.chictr.org.cn/showproj.html?proj=188270, identifier: ChiCTR2200067178.

## 1 Introduction

Severe acute respiratory syndrome coronavirus (SARS-CoV-2) was first identified as the cause of coronavirus disease 2019 (COVID-19) at the end of 2019. COVID-19 rapidly spread globally owing to its high transmissibility and significant morbidity and mortality rates, becoming one of the major public health challenges of our time. Severe cases and mortality rates have been significantly controlled over the past 2 years through vaccination, public health interventions, and the use of antiviral medications; nevertheless, the disease remains incomplete. The Omicron variant and its derived strains are currently dominant, primarily causing mild-to-moderate symptoms in clinical settings (1). In late 2022, the Omicron strain rapidly disseminated in China, infecting ~80% of the population (2). During this period, fever emerged as an initial symptom for the majority of infected individuals, with over 80%−90% of patients experiencing fever during their illness and most having a body temperature exceeding 38.5°C (2, 3).Despite extensive research on COVID-19, specific antiviral treatment options remain limited, such as nirmatrelvir-ritonavir for mild-to-moderate disease, have resulted in many individuals not receiving timely and effective treatment, exacerbating public health challenges and highlighting the urgent need for alternative and adjunctive therapies (4).

Traditional Chinese medicine (TCM) has developed a mature epidemic management system over its long history of treating infectious diseases and has demonstrated significant efficacy during the COVID-19 pandemic (5). A network meta-analysis of seven types of oral Chinese herbal medicines indicated that TCM-assisted treatment for mild-to-moderate COVID-19 enhanced clinical effectiveness, reduced severe case rates, and significantly alleviated uncomfortable symptoms and laboratory indicators (6). TCM treatment strategies include acupuncture and acupoint applications, in addition to oral herbal medicines. Among these, acupoint applications have garnered increasing attention owing to their simplicity and minimal adverse reactions and may potentially modulate the immune response, relieve symptoms, and promote recovery.

Herbal acupoint application (HAA) combines theories of Chinese medicine, meridians, and acupoints by applying herbal substances to specific acupoints to facilitate transdermal absorption. This method utilizes the regulatory functions of acupoints and herbal medicines to influence the overall function of internal organs. Guidelines for TCM interventions in COVID-19, such as the *Guidance of acupuncture intervention on coronavirus disease 2019 (Second Edition)* (7) and *Expert Consensus on Rehabilitation of Chinese Medicine for COVID-19 (First Edition)* (8), recommend using HAA as a supportive treatment for managing symptoms such as fever, cough, and fatigue in patients with mild-to-moderate disease. A multicenter retrospective study on patients with exogenous fever demonstrated significant antipyretic effects of HAA, notably reducing the duration until the first disappearance of fever symptoms; moreover, patients receiving HAA experienced a 1.82-fold higher rate of symptom resolution than those who did not receive treatment (9). Furthermore, a real-world study involving 1.23 million cases demonstrated that HAA could reduce antibiotic usage rates among patients with fever and shorten fever duration (10). Nonetheless, rigorous clinical evidence supporting the efficacy and safety of HAA in respiratory infections, particularly COVID-19, remains insufficient.

Therefore, this multicenter, randomized, double-blind, placebo-controlled clinical trial was conducted to evaluate the effectiveness and safety of HAA in adult patients with fever and mild-to-moderate COVID-19.

## 2 Methods

### 2.1 Study design

This multicenter, randomized, double-blind, placebo-controlled clinical study was conducted in six hospitals in Beijing and Shanxi Province, China. The research protocol was approved by the Clinical Research Ethics Committees (Ethical Approval ID: 2022-271-KY). This study was registered in the Chinese Clinical Trial Registry (https://www.chictr.org.cn, registration number: ChiCTR2200067178) and was conducted in accordance with the Declaration of Helsinki and Good Clinical Practice guidelines. All participants provided informed consent.

### 2.2 Participants

Febrile patients infected with COVID-19 were recruited from hospitals and communities through websites and posters during the COVID-19 outbreak from December 2022 to January 2023. All enrolled patients ① met the following criteria: patients who met the diagnostic criteria for COVID-19; ② clinical classification was mild, moderate type; ③ the first onset of symptoms (or confirmed disease) to the random medication was not more than 48 h, and treatment was not administered; ④ body temperature ≥37.3°C; ⑤ aged 18–65 years, with no limits regarding gender; and ⑥ voluntarily participated in this clinical trial, gave informed consent, and signed the informed consent form.

Patients were excluded in the presence of any of the following conditions: ① bacteria, fungi, or infections other than novel coronavirus infection; ② severe primary diseases such as cardiovascular, cerebrovascular, liver, kidney, hematopoietic, endocrine system, or immune system diseases (upper limit of liver function ALT and AST > normal reference value, the upper limit of serum creatinine > normal reference value, or poor blood glucose control); ③ cognitive disability or psychiatric diseases; ④ planned pregnancy, pregnancy, or lactating during the trial; ⑤ allergy constitution or allergy to the drug ingredients and excipients of this test; ⑥ participated in other clinical trials in the recent 1 month; or ⑦ deemed inappropriate to participate in this clinical trial by the investigator.

### 2.3 Randomization and blinding

In this study, the appropriate block size was selected using SAS 9.4 statistical software, and a randomization sequence was generated using a random seed. Eligible participants were randomly assigned to either the herbal or the placebo group in a 1:1 ratio. Personnel not involved in clinical observation, monitoring, or statistical analysis in this trial encoded the herbal paste and placebo based on an established random sequence. Researchers sequentially assigned drug numbers according to the order of participant enrollment, from smallest to largest.

### 2.4 Interventions

Patients in the herbal group received herbal paste applied at the acupoints.

**Figure 1:**
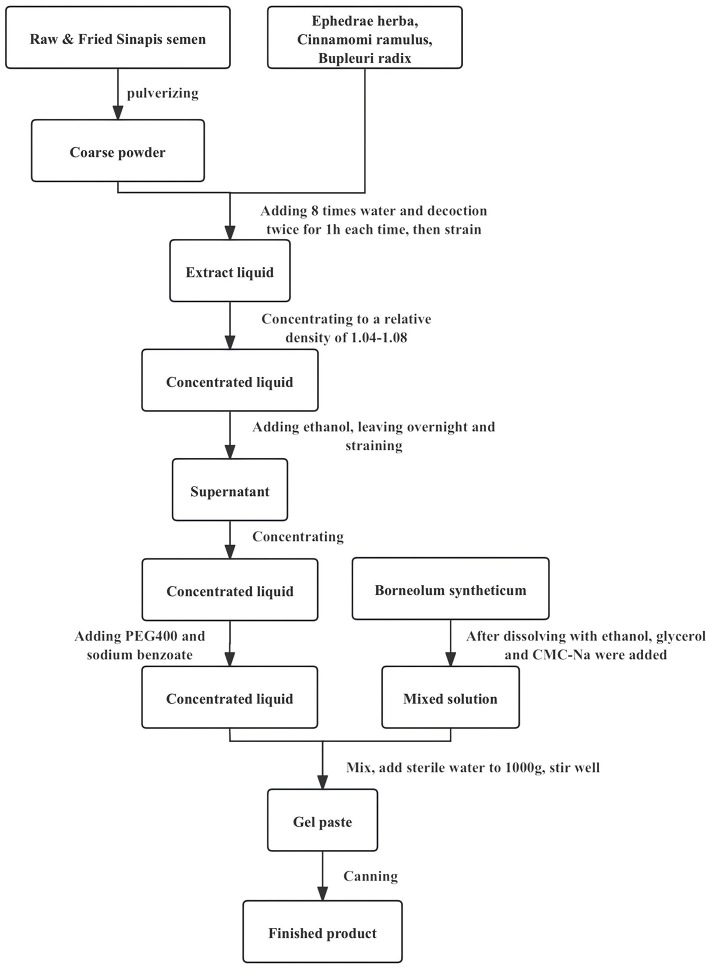
The production process of Chinese herbal acupoint plaster.

The manufacturing process of herbal paste is conducted as follows: The herbal paste comprised *Ephedra sinica* Stapf [Ephedraceae], *Cinnamomum cassia* Presl [Lauraceae], *Bupleurum chinense* DC [Apiaceae], *Sinapis alba* L. [Brassicaceae](Raw), *Sinapis alba* L. [Brassicaceae] (Fried), and Borneolum syntheticum (Table 1). The six herbs were prepared according to the mass ratio of 400:200:200:120:120:9. The raw and fried *Sinapis alba* L. were pulverized and combined with *Ephedra sinica* Stapf, *Cinnamomum cassia* Presl, and *Bupleurum chinense* DC. The mixture was decocted twice using eight volumes of water for 1 h each time, followed by filtration. The filtrate was concentrated to a relative density of 1.04–1.08. Ethanol was then added to achieve a concentration of 60%, thoroughly mixed, and allowed to stand overnight. Following filtration or centrifugation, the supernatant was collected, ethanol was recovered under reduced pressure, and the solution was further concentrated to an appropriate volume. Polyethylene glycol 400 (PEG400) was added, and the mixture was stirred well and set aside. Additionally, Borneolum syntheticum was dissolved in ethanol, followed by the sequential addition of glycerin and carboxymethyl cellulose sodium, stirring thoroughly after each addition. This solution was slowly incorporated into the previously prepared concentrate, then sterile deionized water was added, and the final mixture was thoroughly stirred. Finally, 1 g of herbal paste is approximately equal to 3.487 g of the original medicinal material. The production process of Chinese herbal acupoint plaster is given in Figure 1.

**Table 1:** The composition and processing methods of Chinese herbal acupoint plaster.

**Botanical species**	**Name in Chinese Pharmacopeia**	**Chinese name**	**Plant part**	**Processing method (45)**
*Ephedra sinica* Stapf. [Ephedraceae]	Ephedrae herba	Ma Huang	Stem	Removing woody stems, residual roots and impurities, cutting into segments
*Cinnamomum cassia* Presl. [Lauraceae]	Cinnamomi ramulus	Gui zhi	Twig	Removing impurities, washing, moistening, cutting into thick slices and drying
*Bupleurum chinense* DC. [Apiaceae]	Bupleuri radix	Chai Hu	Root	Removing impurities and stumps, washing, moistening, cutting into thick slices and drying
*Sinapis alba* L. [Brassicaceae]	Sinapis semen (Raw)	Sheng Bai Jie Zi	Seed	Removing impurities
*Sinapis alba* L. [Brassicaceae]	Sinapis semen (Fried)	Chao Bai Jie Zi	Seed	Stir-frying Sinapis semen turn light yellow or dark yellow
/	Borneolum syntheticum	Bing Pian	/	Turpentine was used as the main raw material for artificial synthesis. C_10_H_18_O was not < 55%

The placebo group received a placebo treatment with a similar color, taste, and appearance to the herbal paste used in the herbal group. The appearance difference between placebo and herbal paste is shown in Supplementary Figure S1.

Both groups underwent acupoint application at the same sites using the same methods. According to the recommendations of clinical guidelines (7, 8), the Dazhui (GV14) and bilateral Feishu (BL13) acupoints were chosen for both groups. After routine cleaning and skin disinfection at the acupoints, the herbal or placebo patches were applied to the corresponding acupoints. One gram was used for each acupoint. The patches were applied three times daily, between 6–8 a.m., 1–3 p.m., and 8–10 p.m., for 2 h each time over a 5-day intervention period. If a patient's temperature remained at or below 37.2°C for more than 24 h, they were considered cured, and no further intervention was performed. No antiviral-based treatment was provided to the participants during the study owing to the lack of antiviral medication resources. However, considering ethical factors and after consulting relevant experts, all randomized patients received a basic treatment of the *Fuzheng Jiebiao Decoction* (FZJB), which comprised Astragalus membranaceus [Fabaceae], Atractylodes macrocephala Koidz. [Asteraceae Bercht.], and Saposhnikovia divaricate (Turcz.) Schischk [Apiaceae], administered in three bags per dose, three times a day. If a participant's temperature remained above 39°C for more than 30 min or under other conditions deemed permissible by the attending physician, they were allowed to take one oral tablet of acetaminophen (0.3 g), and the usage was recorded.

### 2.5 Assessments and outcomes

The participants maintained diaries during the trial to log their body temperature, symptom severity, and acetaminophen use. Body temperature was recorded at six intervals daily: 2:00–6:00 a.m., 6:00–10:00 a.m., 10:00 a.m.−2:00 p.m., 2:00–6:00 p.m., 6:00–10:00 p.m., and 10:00 p.m.−2:00 a.m. This monitoring continued until the participant's temperature dropped below 37.3°C for more than 24 h or until the trial concluded. On the fourth and sixth days after treatment initiation, the participants were asked to rate the severity of ten clinical symptoms on a scale from 0 to 10, with higher scores indicating greater severity (0 representing no symptoms and 10 the most severe symptoms): fever, weakness, cough, body aches, taste, and smell abnormalities, diarrhea, nasal congestion, runny nose, headache, and fatigue (11). Laboratory evaluations including complete blood counts, liver and kidney function tests, urinalysis, and electrocardiography were performed at baseline and again on the sixth day after treatment initiation.

The primary outcome measure was complete fever relief time, defined as the duration from the first dose after randomization until the body temperature remained at or below 37.2°C, without subsequent elevation throughout the study period. Secondary outcomes included the onset time of fever reduction (the time from the first dose after randomization to the first drop in temperature to 37.2°C), changes in total symptom scores and individual symptom scores from baseline to the fourth and sixth days post-treatment, usage rate and dosage of acetaminophen, and changes in blood routine tests from baseline to the sixth day. Safety outcomes involved monitoring vital signs, laboratory test results, electrocardiograms, and frequency and severity of adverse events (AEs).

### 2.6 Statistical analysis

The sample size for this study was calculated based on the complete fever relief time in patients with fever due to COVID-19 as the primary efficacy endpoint. According to preliminary observations, the median complete fever relief time for patients with fever caused by mild-to-moderate COVID-19 is 72 h. Assuming that HAA can shorten the complete fever relief time to 48 h, a one-sided log-rank test was employed with a test power (1-β) of 90% and a one-sided false positive rate (α) of 2.5%. Patients were allocated in a 1:1 ratio between the two groups. Considering a 10% dropout and exclusion rate during the study, the final sample size was calculated to be 364 patients, with 182 patients in the herbal and placebo groups.

All participants who were randomized and received at least one dose of the study medication were included in the full analysis set (FAS), in accordance with the intention-to-treat principle. Within the FAS, participants who adhered to the treatment protocol, demonstrated good compliance, completed the primary efficacy endpoint, and did not use any concomitant medications that could significantly impact efficacy assessment or exhibit major protocol deviations were included in the per-protocol set (PPS). The safety analysis set (SS) comprised all randomized participants who received at least one dose of the study medication and had available safety evaluations. SAS (version 9.4) was used to perform statistical analysis. The mean ± standard deviation and median (Q1, Q3) were used to describe continuous variables, and frequencies were used for categorical variables. The *t*-test or Mann–Whitney *U* test was used to compare continuous variables, and the chi-square test or Fisher's exact test was used to compare categorical variables between the groups. Both primary and secondary outcomes were analyzed using the FAS and PPS. The complete fever relief time and onset time of fever reduction were calculated using Kaplan–Meier estimates to determine the median time from dosing to the endpoint event, with hazard ratio (HR) and 95% confidence intervals (CI). Event rates at various time points following treatment initiation were calculated, Kaplan–Meier curves were plotted, and the log-rank test was used to compare intergroup differences. Statistical significance was set at *p*-value < 0.05.

## 3 Results

### 3.1 Study population

**Figure 2:**
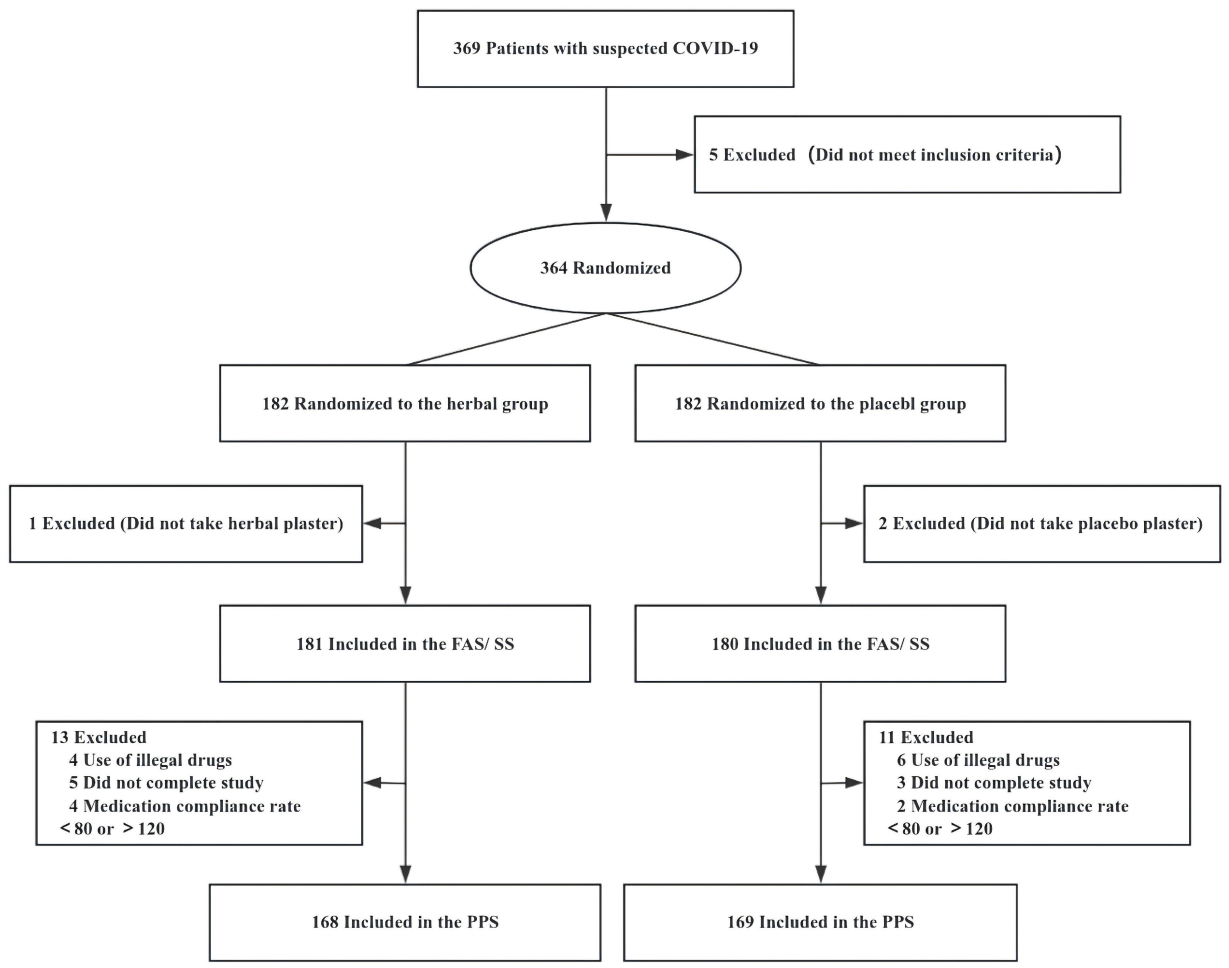
Study flow diagram. FAS, full analysis set; PPS, per-protocol set; SS, safety analysis set.

Overall, 369 patients were screened across six hospitals in China from December 23, 2022, to January 28, 2023. Of these, 364 met all inclusion criteria and none of the exclusion criteria and were randomly assigned to receive either HAA (*n* = 182) or placebo acupoint applications (*n* = 182). Three patients were excluded from the FAS and SS because they did not use the study medication. Of the remaining 361 participants, 337 completed the study and were included in the PPS; 10 participants used prohibited medications, eight withdrew early, and six did not meet the compliance requirement of 80%−120% for medication adherence (Figure 2).

The baseline demographic and clinical characteristics of the FAS were comparable between the herbal paste and placebo group, except for body aches, which differed between the groups (Table 2). The median age of the participants was 33 years (28, 42.5), and 42.66% of the sample were male. Across both groups, 90.86% of participants presented with mild COVID-19, with a median temperature of 38.0°C (37.8, 38.4) and a mean disease duration of 22.79 ± 10.08 h, and 8.03% of participants had comorbid conditions. The median total symptom score (out of a maximum of 100 points) was 34.0 (25.0, 42.5) in the herbal group and 35.0 (27.0, 44.0) in the placebo group. However, the body ache score was significantly higher in the placebo group than in the herbal group (*p* = 0.018).

**Table 2:** Baseline demographic and clinical characteristics of the FAS.

**Variables**	**Herbal group (*n* = 181)**	**Placebo group (*n* = 180)**	**Total (*n* = 361)**	***t/Z/χ^2^***	***p*-Value**
Age (years)	34 (28, 43)	33 (28, 42)	33 (28, 42.5)	−0.214	0.831
**Sex**				0.067	0.796
Men	76 (41.99)	78 (43.33)	154 (42.66)		
Women	105 (58.01)	102 (56.67)	207 (57.34)		
**Race**				0.286	0.593
Han	173 (95.58)	174 (96.67)	347 (96.12)		
Other	8 (4.42)	6 (3.33)	14 (3.88)		
BMI (kg/m^2^)	23.15 (20.90, 26.03)	22.75 (20.52, 25.35)	22.89 (20.70, 25.93)	−0.754	0.451
Comorbid conditions	15 (8.29)	14 (7.78)	29 (8.03)	0.032	0.859
Allergic history	4 (2.21)	6 (3.33)	10 (2.77)	0.423	0.516
Course of disease (hours)^a^	22.94 ± 9.83	22.64 ± 10.35	22.79 ± 10.08		0.775
**Clinical classification**				0.028	0.868
Mild	164 (90.61)	164 (91.11)	328 (90.86)		
Moderate	17 (9.39)	16 (8.89)	33 (9.14)		
**Vital sign**	
Body temperature (°C)	38.1 (37.8, 38.4)	38.0 (37.8, 38.4)	38.0 (37.8, 38.4)	−0.687	0.492
RR (times per minute)	19.0 (18.0, 20.0)	19.0 (18.5, 20.0)	19.0 (18.0, 20.0)	−1.580	0.564
HR (times per minute)	80 (71, 86)	76 (70, 85)	78.0 (70.0, 85.5)	−0.577	0.114
SBP (mmHg)	118.0 (110.0, 125.0)	118.5 (110.0, 123.0)	118.0 (110.0, 124.0)	−0.720	0.472
DBP (mmHg)	79 (74, 83)	78 (73, 82)	79 (74, 83)	−1.289	0.197
**Symptom score**	
Total scores^b^	34.0 (25.0, 42.5)	35.0 (27.0, 44.0)	34.0 (26.0, 43.0)	−0.945	0.345
Fever	6 (5, 8)	6 (5, 8)	6 (5, 8)	−0.120	0.904
Weakness	5 (4, 6)	5 (4, 7)	5 (4, 7)	−1.588	0.112
Cough	2 (1, 5)	3 (1, 5)	3 (1, 5)	−0.219	0.827
Body aches	5 (3, 6)	5 (4, 7)	5 (3, 7)	−2.358	0.018
Taste/smell abnormalities	0 (0, 3)	1 (0, 3)	0 (1, 3)	−1.597	0.110
Diarrhea	0 (0, 1)	0 (0, 1)	0 (0, 1)	−0.225	0.822
Nasal congestion	2 (0, 4)	2 (0, 4)	2 (0, 4)	−0.460	0.645
Runny nose	1 (0, 3)	2 (0, 3)	1 (0, 3)	−0.034	0.973
Headache	4 (2, 6)	4 (2, 6)	4 (2, 6)	−0.362	0.717
Fatigue	5.0 (4.0, 6.5)	5.0 (3.0, 7.0)	5.0 (3.0, 7.0)	−1.127	0.260
**Blood routine tests**	
RBC (10^12^/L)	4.64 ± 0.51	4.68 ± 0.53	4.66 ± 0.52	0.600	0.549
WBC (10^9^/L)	6.48 ± 1.77	6.61 ± 1.95	6.55 ± 1.86	0.683	0.495
HGB (g/L)	140.0 (132.5, 154.0)	141.0 (127.0, 156.0)	140.0 (130.0, 155.0)	−0.359	0.720
PLT (10^9^/L)	251.0 (208.0, 292.0)	249.0 (201.0, 293.0)	251.0 (203.5, 292.5)	−0.491	0.623
NEUT %	60.08 ± 9.27	60.13 ± 9.65	60.11 ± 9.45	0.961	0.961
LYMPH %	31.42 ± 8.93	31.5 ± 9.28	31.47 ± 9.10	0.925	0.925
CRP (mg/L)	2.88 (1.03, 10.00)	1.92 (0.77, 9.73)	2.07 (0.90, 10.00)	−1.212	0.226

Normally distributed data are expressed as mean ± standard deviation; otherwise, the median (Q1, Q3) is expressed. Categorical variables are expressed as n (%).

BMI, body mass index; RR: respiratory rate; HR: heart rate; SBP, systolic blood pressure; DBP, diastolic blood pressure; RBC, red blood cell count; WBC, white blood cell count; HGB, hemoglobin; PLT, platelet count; NEUT %, neutrophil percentage; LYMPH %, lymphocyte percentage; CRP, C-reactive protein.

^a^Course of the disease was defined as the time from symptom onset to the first dose after randomization.

^b^The sum of 10 individual symptom scores, ranging from 0 to 100.

### 3.2 Primary and secondary outcomes

**Figure 3:**
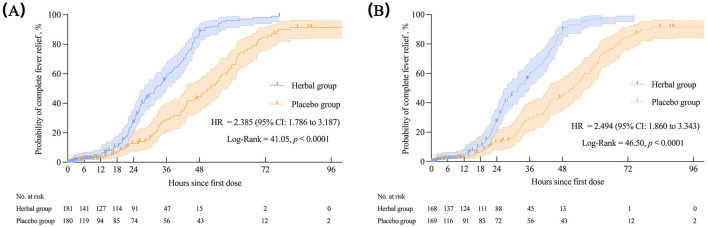
The complete fever relief time in the FAS **(A)** and PPS **(B)**. Kaplan–Meier was used to estimate the median complete fever relief time, with HR and 95%CI. The log-rank test was used to compare inter-group differences. FAS, full analysis set; PPS, per-protocol set; HR, hazard ratio; CI, confidence interval.

Regarding the primary outcome, the median complete fever relief time in the FAS was 31.75 h in the herbal group and 52.00 h in the placebo group, with statistically significant differences between the groups [*p* < 0.0001, HR = 2.385 (95% CI: 1.786–3.187)]. Similar results were observed for the PPS. The Kaplan–Meier survival curves for the FAS and PPS are shown in Figure 3.

**Figure 4:**
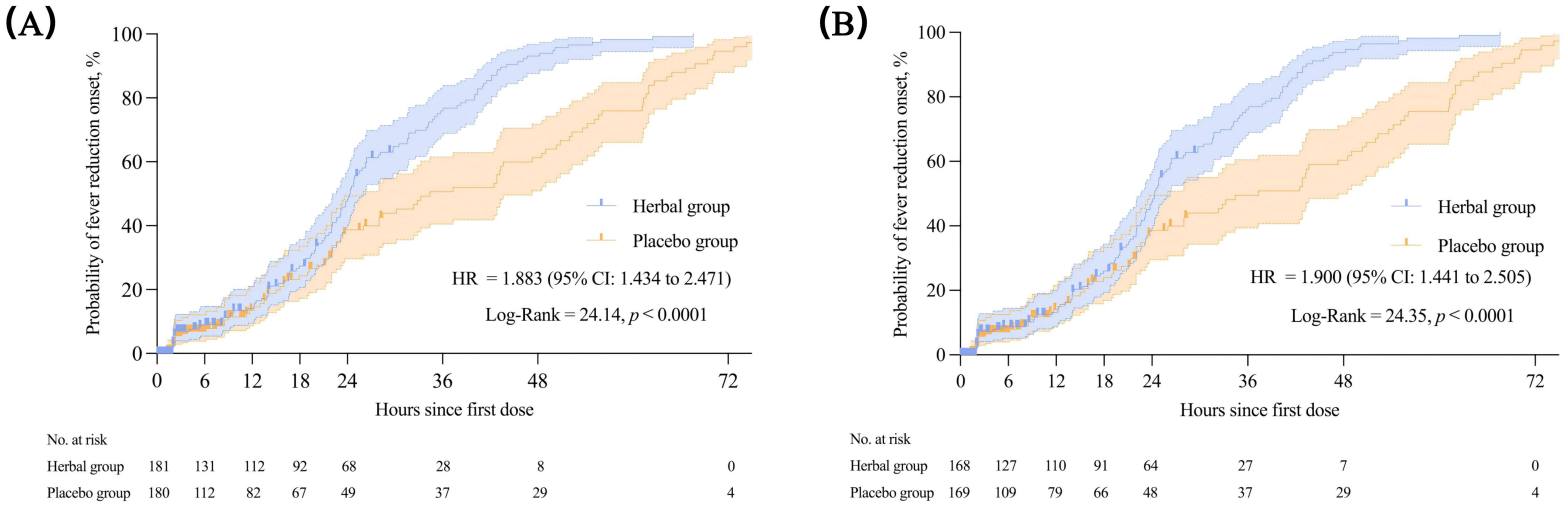
The onset time of fever reduction in the FAS **(A)** and PPS **(B)**. Kaplan–Meier was used to estimate the median onset time of fever reduction, with HR and 95% CI. The log-rank test was used to compare inter-group differences. FAS, full analysis set; PPS, per-protocol set; HR, hazard ratio; CI, confidence interval.

The herbal group had a shorter onset time of fever reduction than the placebo group (24.35 vs. 34.42 h), with statistically significant differences between the two groups demonstrated by the log-rank test [*p* < 0.0001, HR = 1.883 (95% CI: 1.434–2.471)]. The Kaplan–Meier survival curves for the FAS and PPS are shown in Figure 4. When compared with the baseline, the median total symptom score changed by −17 (−23, −10) in the herbal group and −12 (−21, −7) in the placebo group on the fourth day of treatment, with a significant difference between the two groups (*p* = 0.004). Regarding the change from the baseline in individual symptom scores on the fourth day after treatment, fever (−6[−7, −5] vs. −5[−7, −4], *p* < 0.001), body aches (−3 [−5, −2] vs. −3 [−4, −1], *p* = 0.019), and headache (−3[−4, −1] vs. −2[−3, −1], *p* = 0.048) were reduced to a greater extent in the herbal group. Cough (0.30 ± 2.63 vs. 0.89 ± 2.87) increased to a lesser degree in the herbal group (*p* = 0.036; Table 3). No significant disparities were observed in weakness, taste and smell abnormalities, diarrhea, nasal congestion, runny nose, and fatigue scores on the fourth day of treatment or in the total score and the scores of each symptom on the sixth day of treatment (*p* > 0.05; Table 3). The results of the PPS were analogous to those of the FAS, except for alterations in cough and headache on the fourth day of treatment between the herbal and placebo groups (Supplementary Table S1). Regarding acetaminophen use, 52 patients (29.05%) in the herbal group used acetaminophen, which was lower than the 94 patients in the placebo group (*p* < 0.001). Additionally, inter-group disparities were observed in acetaminophen dosage [herbal group: 0.3 (0.3, 0.6) g vs. placebo group: 0.6 (0.6, 0.9) g; *p* < 0.01; Table 3]. No significant differences were observed in routine blood tests between the herbal and placebo groups (*p* > 0.05; Table 3).

**Table 3:** Primary and secondary outcomes (FAS).

**Outcomes**	**Herbal group (*n* = 181)**	**Placebo group (*n* = 180)**	***Z/ χ^2^***	***p-*Value**
**Primary outcome**			
Complete fever relief time (h)	31.75	52.00	–	< 0.0001
**Secondary outcomes** ^a^			
Onset time of fever reduction (h)	24.35	34.42	–	< 0.0001
**Change of symptom score**			
**Total scores**			
4-day	−17 (−23, −10)	−12 (−21, −7)	−2.848	0.004
6-day	−24 (−33, −16)	−22 (−34, −14)	−0.661	0.508
**Fever**			
4-day	−6 (−7, −5)	−5 (−7, −4)	−3.605	< 0.001
6-day	−6 (−8, −5)	−6.00 (−8, −5)	−0.214	0.831
**Weakness**			
4-day	−2.0 (−3.0, 0.0)	−1.5 (−3.0, 0.0)	−1.437	0.151
6-day	−3 (−5, −2)	−3 (−5, −2)	−0.067	0.946
**Cough**			
4-day	0 (−1, 2)	1 (−1, 3)	−2.093	0.036
6-day	−1 (−3, 2)	0 (−2, 3)	−1.653	0.098
**Body aches**			
4-day	−3 (−5, −2)	−3 (−4, −1)	−2.354	0.019
6-day	−4 (−6, −3)	−4 (−6, −3)	−0.713	0.476
**Taste/smell abnormalities**			
4-day	0 (−1, 0)	0 (−1, 0)	−0.084	0.933
6-day	0 (−2, 0)	0 (−3, 0)	−0.316	0.752
**Diarrhea**			
4-day	0 (0, 0)	0 (0, 0)	−0.578	0.563
6-day	0 (−1, 0)	0 (0, 0)	−0.288	0.773
**Nasal congestion**			
4-day	−1 (−2, 0)	0 (−2, 1)	−1.375	0.169
6-day	−2 (−3, 0)	−1 (−3, 0)	−1.019	0.308
**Runny nose**			
4-day	0 (−2, 1)	0 (−2, 1)	−0.050	0.960
6-day	−1 (−2, 0)	−1 (−3, 0)	−0.177	0.860
**Headache**			
4-day	−3 (−4, −1)	−2 (−3, −1)	−1.981	0.048
6-day	−3 (−5, −2)	−4 (−5, −2)	−0.163	0.870
**Fatigue**			
4-day	−2 (−3, 0)	−1 (−3, 0)	−1.381	0.167
6-day	−3 (−5, −1)	−3 (−5, −1)	−0.118	0.906
**Acetaminophen use**			
*n* (%)	52 (29.05)	94 (52.22)	19.972	< 0.001
Dosage (g)^b^	0.3 (0.3, 0.6)	0.6 (0.3, 0.9)	−4.256	< 0.001
**Change of blood routine tests, 6 days from baseline**			
RBC (10^12^/L)	−0.01 (−0.17, 0.16)	−0.03 (−0.22, 0.13)	−1.336	0.181
WBC (10^9^/L)	−0.23 (−1.24, 0.73)	−0.14 (−1.12, 0.70)	−0.129	0.897
HGB (g/L)	−1 (−4, 4)	−1 (−6, 3)	−0.915	0.360
PLT (10^9^/L)	−13.0 (−39.5, 12.0)	−10.0 (−31.0, 19.0)	−1.043	0.297
NEUT%	0.30 (−5.75, 4.55)	−0.70 (−5.50, 4.40)	−0.521	0.603
LYMPH%	−0.10 (−4.00, 4.95)	−0.10 (−4.10, 6.20)	−0.639	0.524
CRP (mg/L)	−0.10 (−4.10, 0.50)	−0.02 (−1.31, 0.13)	−0.515	0.607

Normally distributed data are expressed as mean ± standard deviation; otherwise, the median (Q1, Q3) is expressed. Categorical variables are expressed as n (%).

RBC, red blood cell count; WBC, white blood cell count; HGB, hemoglobin; PLT, platelet count; NEUT %, neutrophil percentage; LYMPH %, lymphocyte percentage; CRP, C-reactive protein; FAS, full analysis set.

^a^Missing data not imputed for secondary outcomes analyses.

^b^Number of participants: 52 in the herbal group and 94 in the placebo group.

### 3.3 Safety analysis

Overall, 73 patients (20.22%) reported 87 AEs during the 6-day follow-up period, with 35 (19.34%) in the herbal group and 38 (21.11%) in the placebo group. Among them, 34 patients (9.42%) reported 44 AEs related to the study medication [19 (10.50%) in the herbal group and 15 (8.33%) in the placebo group], all of which were reactions at the application sites. These included itching in 25 cases (14 in the herbal group and 11 in the placebo group), erythema in nine cases (five in the herbal group and four in the placebo group), increased skin temperature in six cases (five in the herbal group and one in the placebo group), and rash in four cases (one in the herbal group and three in the placebo group). No serious adverse events (SAEs) or unexpected serious adverse reactions were observed in either group. In addition to the reported AEs, no notable abnormalities were found on physical examination, vital signs, laboratory test results, or electrocardiogram results after treatment (Table 4).

**Table 4:** Safety analysis (SS).

**Adverse events**	**Herbal group (*n* = 181)**	**Placebo group (*n* = 180)**
**All adverse events**	35 (19.34)	38 (21.11)
Serious adverse events	0 (0.00)	0 (0.00)
Adverse events leading to dropout	1 (0.55)	1 (0.56)
**Drug-related adverse events**	19 (10.50)	15 (8.33)
Itch at application site	14 (7.73)	11 (6.11)
Erythema at application site	5 (2.76)	4 (2.22)
Increased skin temperature at application site	5 (2.76)	1 (0.56)
Rash at application site	1 (0.55)	3 (1.67)
Serious drug-related adverse events	0 (0.00)	0 (0.00)
Drug-related adverse events leading to dropout	0 (0.00)	1 (0.56)

Data are expressed as n (%). Adverse events in this study were collected through the active reporting of subjects, oral questions from researchers, and a combined drug review. All adverse events were graded according to the Common Terminology Criteria for Adverse Events (CTCAE) v5.0.

## 4 Discussion

An increasing number of high-quality randomized controlled trials focusing on the clinical efficacy of TCM in treating COVID-19 have confirmed the significant benefits of oral Chinese herbal medicine (4, 12). HAA for COVID-19 has been widely recommended by clinical guidelines in China; however, high-quality evidence supporting its efficacy remains lacking. To address this gap, we conducted a multicenter, randomized, double-blind, placebo-controlled clinical study to evaluate the efficacy and safety of HAA. The results indicated that HAA combined with oral FZJB significantly accelerates defervescence and symptom relief in adults with mild-to-moderate COVID-19, notably reduces the need for antipyretic analgesics, and demonstrates a favorable safety profile.

Fever is a hallmark of infectious and inflammatory diseases (13). In COVID-19, the coronavirus enters cells by binding to the host cell receptor angiotensin-converting enzyme 2 (ACE2) and the cellular serine protease TMPRSS2, triggering immune system activation, and an inflammatory response (14, 15). This inflammatory reaction releases pyrogenic cytokines, such as interleukin (IL)-1, IL-6, and tumor necrosis factor (TNF), which stimulate the hypothalamic temperature regulation center and raise the body's temperature set point (16). The Omicron variant generally causes “mild infection,” with lower rates of hospitalization and mortality compared to the Delta variant (17–19). According to data from the ZOE COVID study, fever was less frequently reported during the omicron variant surge (20). However, in China, data from the same period as our study indicate that fever remains one of the most commonly reported symptoms (2, 3). Among our study participants, 185 (50.82%) had mild fevers (37.3–38°C), 164 (45.06%) had moderate fevers (38.1–39°C), and 15 (4.12%) had high or very high fevers (over 39°C). In contrast to other studies (2, 3), the majority of participants in our study had temperatures below 38°C, which may be associated with the early stage of disease intervention, as the average time from symptom onset to treatment was < 1 day (22.79 ± 10.08 h, Table 2). Moreover, fever constitutes an adaptive response to infection, with elevated body temperatures potentially enhancing the immune response against infection (13). One study showed that fever may inhibit SARS-CoV-2 replication in respiratory epithelial cells (21). Consequently, studies have recommended against the use of antipyretic analgesics (22). However, no clear evidence exists that links antipyretic analgesic use to adverse COVID-19 outcomes (23, 24). Similarly, the use of these medications in other infectious diseases remains controversial (25, 26).

To the best of our knowledge, this was the first multicenter, randomized, double-blind, placebo-controlled trial that evaluated the efficacy and safety of HAA for the treatment of fever and other symptoms associated with COVID-19. This study demonstrated clinically significant antipyretic effects of HAA. Patients treated with HAA had a shorter complete fever relief time (31.75 vs. 52.00 h) and a shorter onset time of fever reduction (24.35 vs. 34.42 h) than the placebo group. Additionally, fewer participants in the herbal group required acetaminophen (52 vs. 92), and the average dosage was lower [0.3 g (0.3, 0.6) vs. 0.6 g (0.3, 0.9)]. HAA showed superior efficacy in alleviating overall clinical symptoms compared with placebo, particularly fever, headache, and cough. A difference in body ache scores was observed on the fourth day of treatment between the two groups; however, these results were not comparable because the placebo group had notably higher baseline scores than the herbal group. These findings suggest a potential effect of HAA in relieving body aches; nevertheless, further research is required to confirm this effect. HAA showed no significant advantages in improving blood test parameters.

To date, no study has specifically examined the antipyretic mechanisms of HAA. Nevertheless, it is likely that the combined effect of the herbal ingredients and acupoint stimulation contributed to the therapeutic efficacy observed in this treatment. The herbal formulas used in this study included Ephedra, Bupleurum, cinnamon twig, Borneol, and white mustard seed, which were applied to acupoints GV14 and BL13. These herbs and acupoints are widely believed to promote sweating, relieve surface-level infections, detoxify, and alleviate respiratory symptoms. Therefore, this treatment is suitable for managing fever and other related symptoms in patients with COVID-19. Network pharmacology has shown that Ephedra and cinnamon twigs, commonly paired for their diaphoretic and antipyretic properties, target multiple biological processes and pathways (27). This combination may function synergistically to inhibit inflammation, reduce viral activity, and protect tissues, thereby alleviating the cytokine storm associated with COVID-19 fever. When applied to the GV14, Ephedra and cinnamon twig are believed to reduce the expression of fever-inducing cytokines such as IL-6, IL-1β, and TNF-α in lung tissue, which may stabilize fever (28). Bupleurum, a traditional antipyretic herb, exerts anti-inflammatory, antiviral, and immunoregulatory effects (29). It directly suppresses TNF-α production by monocytes and inhibits the complement system, lowering circulating TNF-α levels to exert antipyretic effects (30). Borneol is an effective penetration enhancer (31, 32). Studies have shown that it disrupts the ordered arrangement of lipid matrices in the stratum corneum, facilitating drug absorption (33). Meanwhile, white mustard seed exerts anti-inflammatory effects by downregulating TNF-α and IL-6 mRNA expression (34). However, white mustard seeds can cause blistering as a side effect owing to their active volatile oil (35). This oil has been shown to increase the fluidity and disorder of lipids between stratum corneum cells, promoting drug penetration (36, 37). The GV14 and BL13 acupoints are commonly used to treat respiratory conditions and are among the most frequently targeted points for managing febrile illnesses caused by external pathogens (9). A prospective observational study showed that acupoint stimulation at points including GV14 and BL13 significantly shortened the duration of SARS-CoV-2 RNA positivity, reduced symptom recovery time, and notably alleviated cough, sore throat, and fever compared to basic treatment alone, thereby improving the clinical cure rate (38).

The safety profile of this treatment was promising in this trial. The incidence of AEs was comparable between the herbal paste and the placebo groups, with no severe AEs reported in either group (Table 4). Most AEs resolved over time, suggesting good overall tolerability. Similar to prior studies (39), this study observed skin reactions at application sites, such as itching, erythema, temperature increase, and rash, possibly related to the white mustard seed component or the adhesive materials used.

It is noteworthy that FZJB was used as a standardized background intervention for all participants in this study. This formula is derived from the classical TCM prescription *Yupingfeng San*, which has been widely used in the clinical management of respiratory conditions such as chronic obstructive pulmonary disease (40), respiratory tract infections (41), and pneumonia (42). In the context of COVID-19 prevention and treatment, this decoction is believed to exert adjunctive therapeutic effects by modulating immune function (43). Given the absence of specific antiviral agents during the study period, the inclusion of FZJB was deemed necessary to maintain ethical equipoise. However, although it may lack direct antiviral activity, its potential immunomodulatory effects could confound the assessment of the independent therapeutic efficacy of HAA. As there was no group receiving HAA alone or FZJB alone, it is not possible to determine whether the observed therapeutic effects were due solely to HAA, the decoction, or their synergistic interaction. Future trials with factorial designs or separate arms for each intervention component would allow for clearer attribution of treatment effects. Another important consideration is the limited clinical improvement observed in the placebo group, despite their receiving visually identical acupoint applications as the herbal group. Previous studies on acupoint stimulation have shown that participants' psychological expectations and beliefs regarding the intervention can elicit substantial non-specific placebo responses (44). Unfortunately, this trial did not include assessments of expectancy-related variables, such as perceived treatment credibility, participants' expectations, or their attitudes toward traditional medicine. This methodological limitation may have compromised the accurate interpretation of the between-group differences. Future studies should aim to refine the trial design by incorporating validated expectancy assessment tools to allow a more precise evaluation of the specific therapeutic effects of HAA.

In addition, this study had several limitations. First, although HAA has shown promising clinical efficacy, further investigation to identify the main active ingredients and precise mechanisms of action of herbal formulas. Second, the participants in this study were relatively young; therefore, the effectiveness of the treatment in older patients requires further evaluation. Third, the vaccination status of the participants was not recorded in this study, although previous studies have shown that vaccination can significantly reduce symptom severity and recovery time in COVID-19. Fourth, although body pain showed greater improvement in the herbal group, baseline imbalance prevents definitive attribution of this change to the intervention. Adjusting for baseline scores or applying analysis of covariance in future studies would enhance the robustness of such comparisons. Fifth, no subgroup analysis was conducted to compare the effects of HAA with those of antipyretic drugs, which could offer insights into their efficacy as potential antipyretic agents. Sixth, this study only assessed the short-term outcomes of patients with COVID-19, and the long-term benefits and potential side effects remain unknown. Finally, this study was conducted solely in China, which may limit the generalizability of the results.

## 5 Conclusion

Although all participants were administered the intervention of *Fuzheng Jiebiao Decoction*, the contribution of this decoction to the observed efficacy cannot be entirely disregarded. However, this study still preliminarily indicates that compared with the placebo, Chinese herbal acupoint application effectively and safely shortened the complete fever relief time and onset time of fever reduction; alleviated clinical symptoms, particularly fever, headache, and cough; and reduced the need for antipyretic analgesics in adult patients with fever and mild-to-moderate COVID-19.

## Data Availability

The original contributions presented in the study are included in the article/Supplementary material, further inquiries can be directed to the corresponding authors.
