# Supplementary material for: Effectiveness and safety of Chinese herbal acupoint application in adult patients with fever and mild-to-moderate COVID-19: a multicenter, randomized, double-blind, placebo-controlled trial
Source: Front Neurol. 2025 Jun 19;16:1577976. doi: 10.3389/fneur.2025.1577976 (PMC12223423; doi:10.3389/fneur.2025.1577976)
Supplement: Supplementary file 1 [file Supplementary_file_1.docx]

Supplementary Material

**Effectiveness and Safety of Chinese Herbal Acupoint Application in Adult Patients with Fever and Mild-to-Moderate COVID-19: A Multicenter, Randomized, Double-Blind, Placebo-Controlled Trial**

**Supplementary Figure S1. Difference between Chinese herbal acupoint plaster and placebo**

**
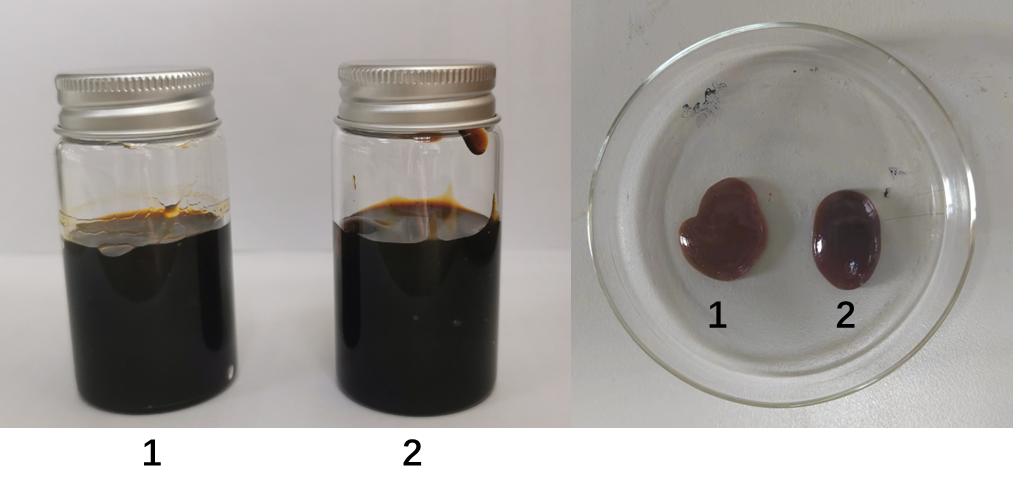
**

1: Chinese herbal acupoint plaster; 2: placebo.

**Supplementary Table S1. Primary and secondary outcomes in PPS**

| **Outcomes** | **Herbal group**  **(*n* = 168)** | **Placebo group**  **(*n* = 169)** | ***Z /χ^2^*** | ***P-*value** |
| --- | --- | --- | --- | --- |
| Primary outcome | | | | |
| Complete fever relief time (h) | 31.75 | 52.00 | - | <.0001 |
| Secondary outcomes ^a^ | | | | |
| Onset time of fever reduction (h) | 24.35 | 34.4 | - | <.0001 |
| Change of symptom score | | | | |
| Total scores | | | | |
| 4-day | -17 (-23, -10) | -13 (-21, -8) | -2.599 | 0.009 |
| 6-day | -25 (-34, -17) | -23 (-34, -15) | -0.653 | 0.516 |
| Fever | | | | |
| 4-day | -6.0 (-7.50, -5.0) | -5.0 (-7.0, -4.0) | -3.618 | ＜.001 |
| 6-day | -6 (-8, -5) | -6(-8, -5) | -0.028 | 0.978 |
| Weakness | | | | |
| 4-day | -2 (-3, 0) | -2 (-3, 0) | -1.320 | 0.187 |
| 6-day | -3 (-5, -2) | -3 (-5, -2) | -0.017 | 0.987 |
| Cough | | | | |
| 4-day | 0 (-1, 2) | 1 (-1 ,3) | -1.920 | 0.055 |
| 6-day | -0.5 (-3.0, 2.0) | 0.0 (-2.0, 2.0) | -1.464 | 0.134 |
| Body aches | | | | |
| 4-day | -3 (-5, -2) | -3 (-4, -1) | -2.554 | 0.011 |
| 6-day | -4 (-6, -3) | -4 (-6, -3) | -0.475 | 0.635 |
| Taste/smell abnormalities | | | | |
| 4-day | 0 (-1, 0) | 0 (-1, 0) | -0.040 | 0.969 |
| 6-day | 0 (-2, 0) | 0 (-3, 0) | -0.147 | 0.884 |
| Diarrhea | | | | |
| 4-day | 0 (0, 0) | 0 (0, 0) | -0.468 | 0.640 |
| 6-day | 0 (0, 0) | 0 (0, 0) | -0.125 | 0.901 |
| Nasal congestion | | | | |
| 4-day | -1(-2, 0) | 0 (-2, 1) | -1.189 | 0.235 |
| 6-day | -2 (-3, 0) | -1 (-3, 0) | -1.041 | 0.298 |
| Runny nose | | | | |
| 4-day | 0 (-2, 0.5) | 0 (-2, 1) | -0.004 | 0.997 |
| 6-day | -1 (-3, 0) | -1 (-3, 0) | -0.122 | 0.903 |
| Headache | | | | |
| 4-day | -3 (-4, -1) | -2 (-3, -1) | -1.70 | 0.061 |
| 6-day | -4.0 (-5.5, -2.0) | -4.0 (-5.0, -2.0) | -0.258 | 0.797 |
| Fatigue | | | | |
| 4-day | -2 (-3, 0) | -1 (-3, 0) | -1.064 | 0.287 |
| 6-day | -3 (-5, -1) | -3 (-5, -1) | 0.000 | 1.000 |
| Acetaminophen use | | | | |
| *n* (%) | 50 (29.76) | 88 (52.07) | 17.340 | <.001 |
| Dosage (g) ^b^ | 0.3 (0.3, 0.6) | 0.6 (0.3, 0.9) | -4.105 | <.001 |
| Change of blood routine tests, 6 days from baseline | | | | |
| RBC (10^12^/L) | 0.00(-0.17, 0.16) | -0.03(-0.22, 0.13) | -1.466 | 0.146 |
| WBC (10^9^/L) | -0.26(-1.27, 0.70) | -0.03(-1.02, 0.71) | -0.384 | 0.692 |
| HGB (g/L) | -1.00(-5.00, 4.00) | -1.00(-6.00, 3.00) | -0.748 | 0.454 |
| PLT (10^9^/L) | -13.00(-40.00, 12.00) | -11.00(-31.00, 21.00) | -1.080 | 0.280 |
| NEUT% | 0.30(-4.90, 4.40) | -0.55(-5.40, 4.90) | -0.328 | 0.748 |
| LYMPH% | 0.10(-3.80, 4.90) | -0.15(-4.10, 5.20) | -0.414 | 0.678 |
| CRP (mg/L) | -0.11(-4.10, 0.50) | -0.08(-1.35, 0.13) | -0.382 | 0.702 |

Normally distributed data are expressed as mean ± standard deviation; otherwise, the median (Q1, Q3) is expressed. Categorical variables are expressed as n (%).

Abbreviations: RBC, Red blood cell count; WBC, white blood cell count; HGB, hemoglobin; PLT, platelet count; NEUT %: neutrophil percentage; LYMPH %: lymphocyte percentage; CRP: C-reactive protein; PPS: per-protocol set.

a Missing data not imputed for secondary outcomes analyses.

b Number of participants: 50 in the herbal group and 88 in the placebo group.
